# Challenges and Opportunities for LGBTQ+ Older Adults in Rural California: A Community-Engaged Focus Group Study

**DOI:** 10.1093/geroni/igaf122.1731

**Published:** 2025-12-31

**Authors:** Angela Perone

**Affiliations:** University of California, Berkeley, Berkeley, California, United States

## Abstract

Research on rural older adults underscores social, health, and economic disparities (Cohen & Greaney, 2022), which mirror many disparities emerging from research on LGBTQ+ older adults (e.g., Fredriksen-Goldsen & Hoy-Ellis, 2017; Lampe et al., 2024; Rowan et al., 2022). Research on LGBTQ+ older adults in rural communities, however, is sparse, and scholars have called for more research on this population (e.g., Guest, Hunger & Schoenberg, 2023; Dakin, Williams, & MacNamara, 2020; Daken, Levy, & Williams, 2022). This community-engaged study incorporates 23 focus groups with 208 LGBTQ+ older adults in California to examine challenges, thriving and surviving strategies, and recommendations regarding health, housing, social services, and caregiving. Community partners collaborated in refining the focus group protocol, recruitment, and dissemination. Of the 23 focus groups, 17 were inter-categorical (included both rural and non-rural participants) (n = 164) and 6 were intra-categorical (included only rural participants) (n = 44). Thematic analysis revealed six interrelated challenges regarding health, housing, technology, transportation, caregiving, and community for LGBTQ+ older adults in rural communities. Informal support networks and community resources also played a vital role in wellbeing and social connectedness. Three themes emerged around policy and practice suggestions to enhance the lives of LGBTQ+ older adults in rural communities: improve access to culturally responsive services, increase opportunities for community gathering spaces, and address transportation barriers and service gaps that may disproportionately impact LGBTQ+ older adults in rural communities. These findings underscore important challenges and opportunities for researchers, policymakers, and practitioners on how to best support LGBTQ+ older adults in rural areas.

